# Supplementary figures and images for: Crystallization of carboplatin-loaded onto microporous calcium phosphate using high-vacuum method: Characterization and release study
Source: PLoS One. 2020 Dec 8;15(12):e0242565. doi: 10.1371/journal.pone.0242565 (PMC7723252; doi:10.1371/journal.pone.0242565)

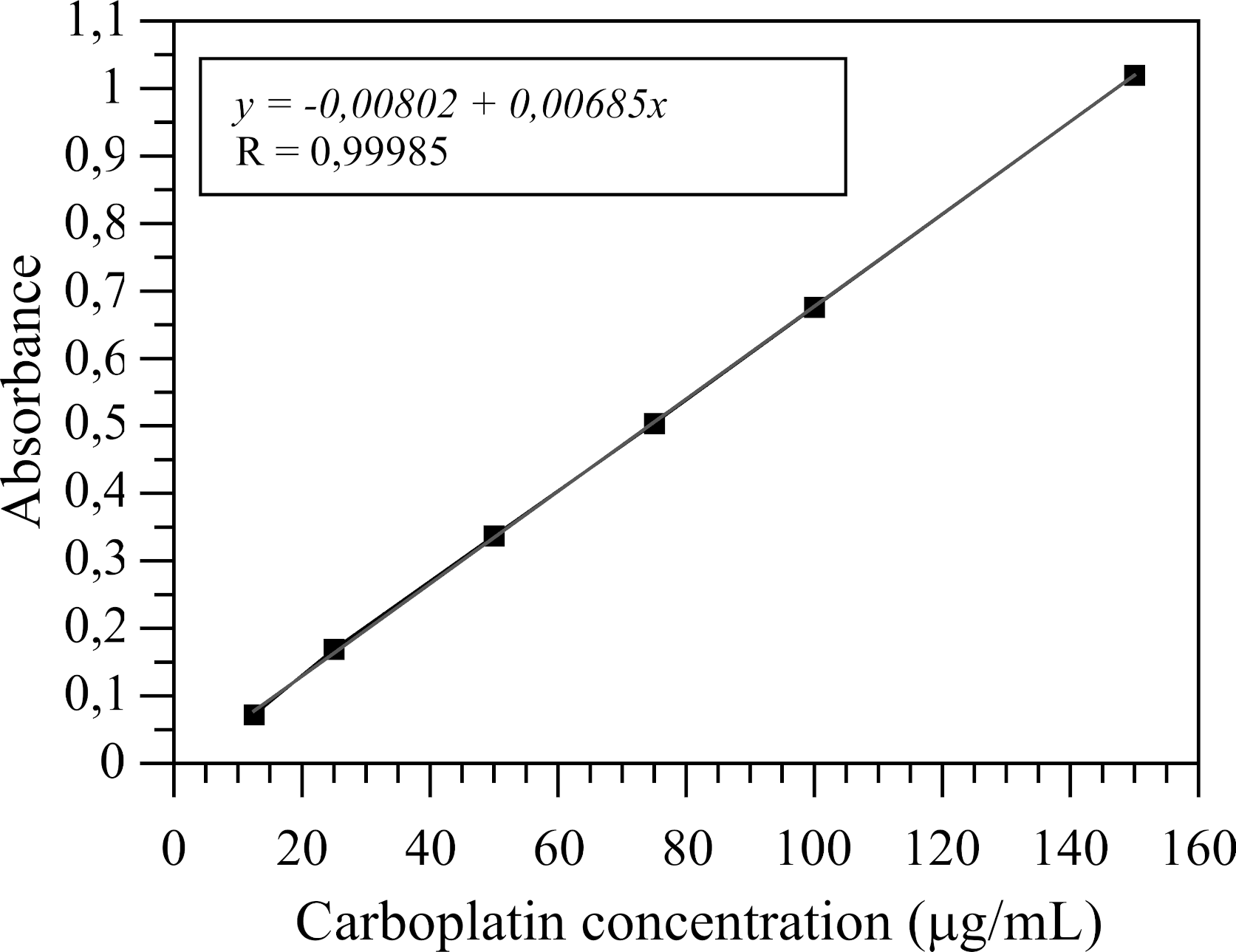

Supplement: S1 Fig — (TIF) [file pone.0242565.s006.tif]

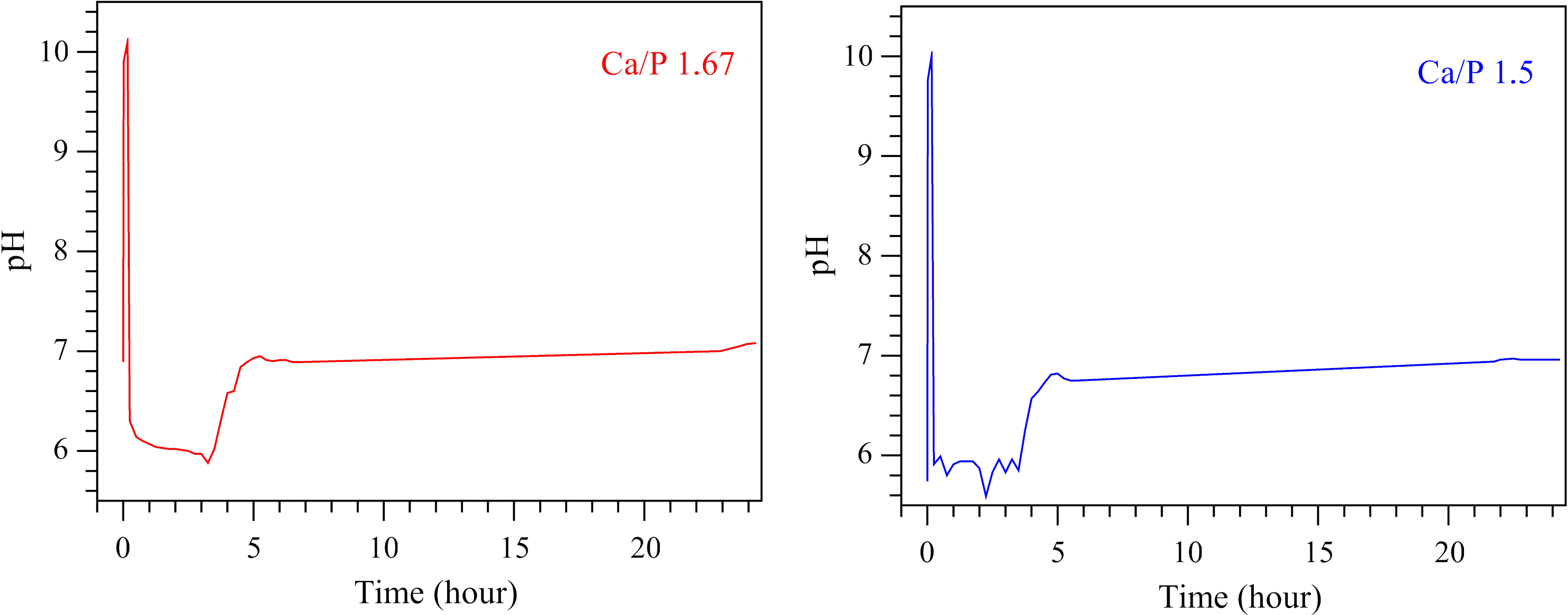

Supplement: S2 Fig — (TIF) [file pone.0242565.s007.tif]

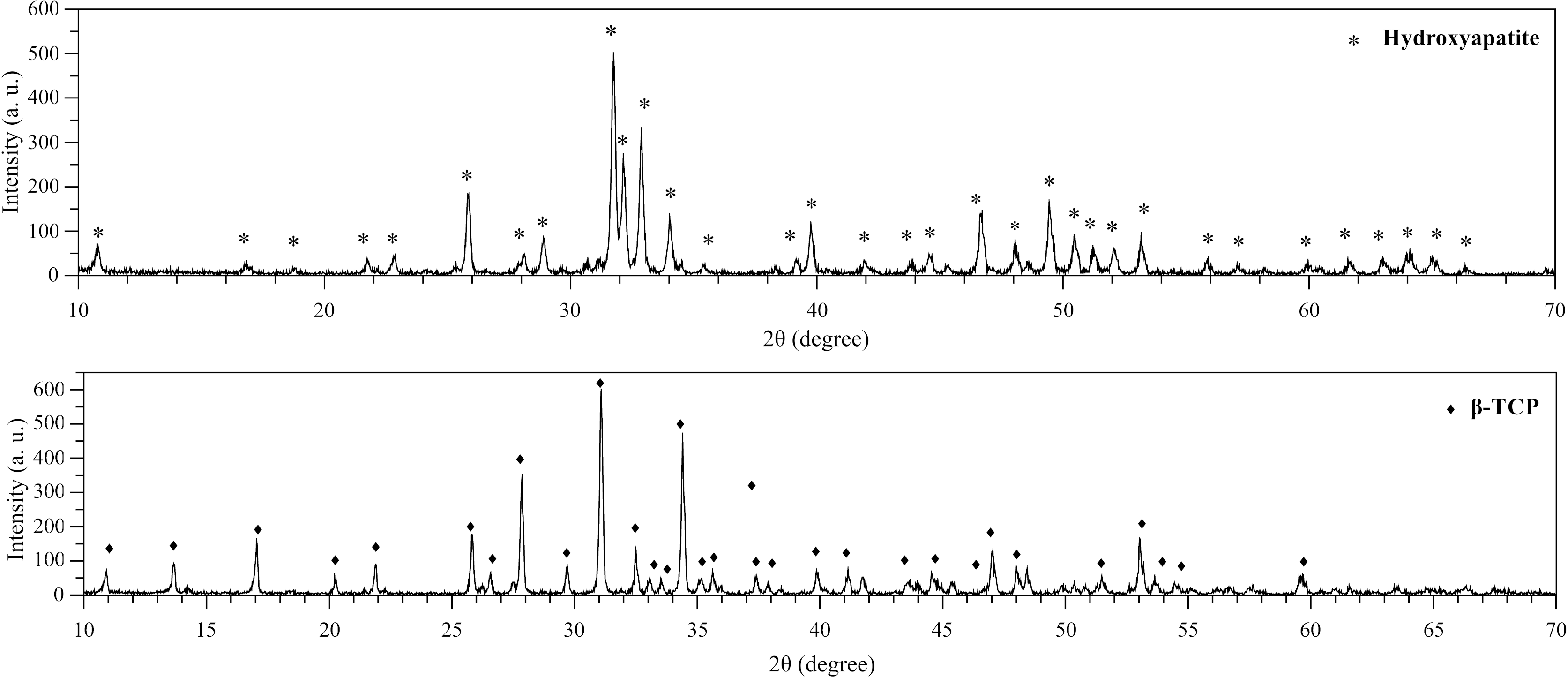

Supplement: S3 Fig — (TIF) [file pone.0242565.s008.tif]

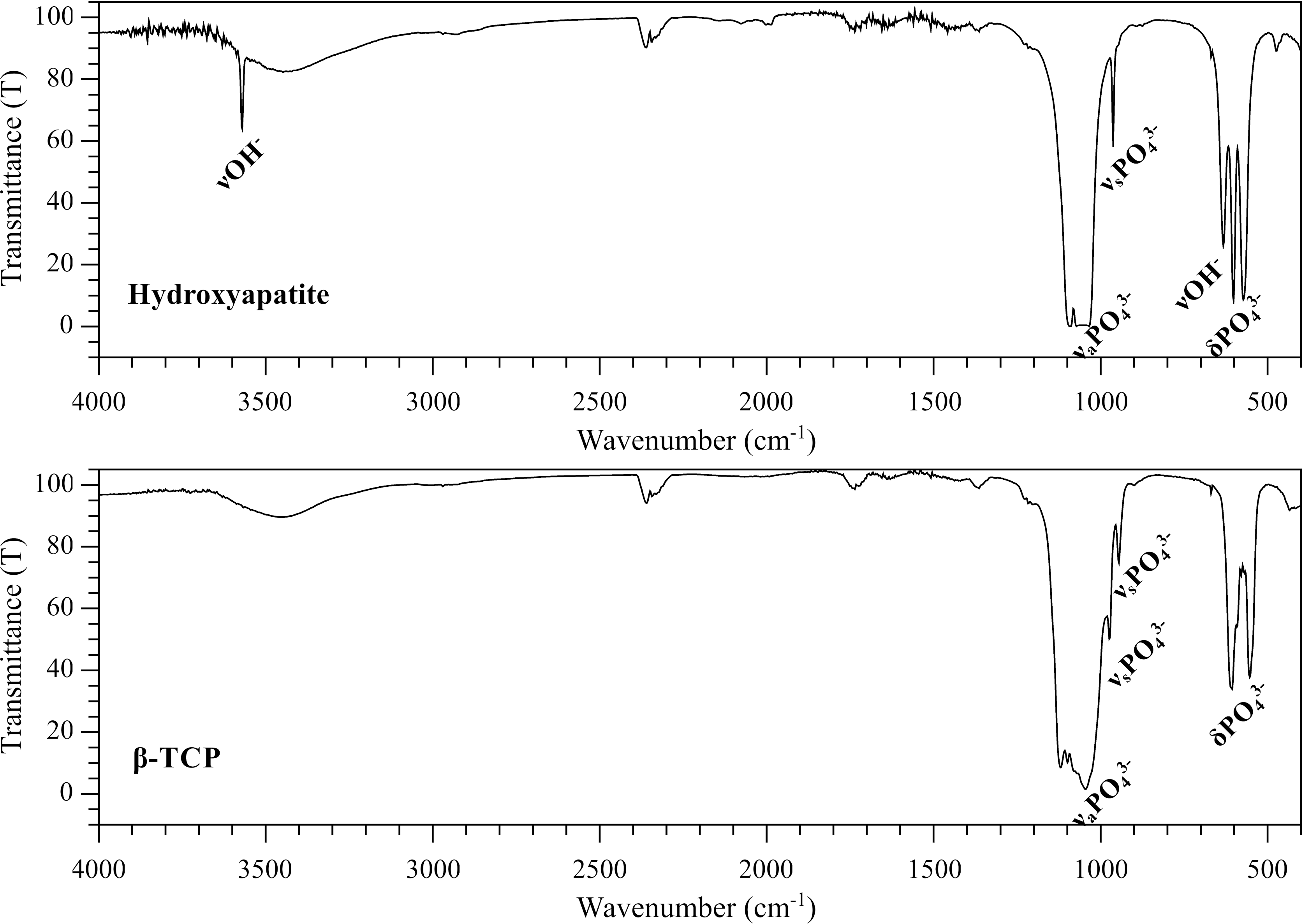

Supplement: S4 Fig — (TIF) [file pone.0242565.s009.tif]

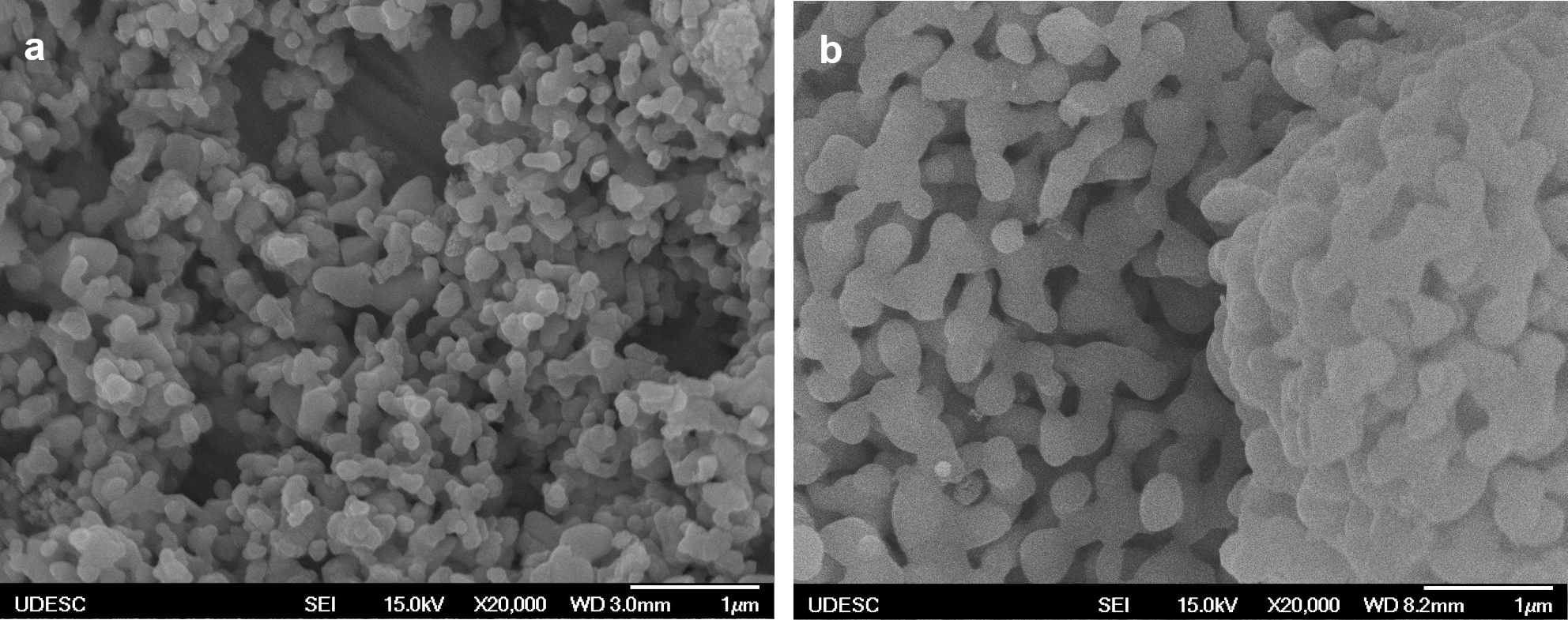

Supplement: S5 Fig — (a) hydroxyapatite and (b) β-TCP. (TIF) [file pone.0242565.s010.tif]

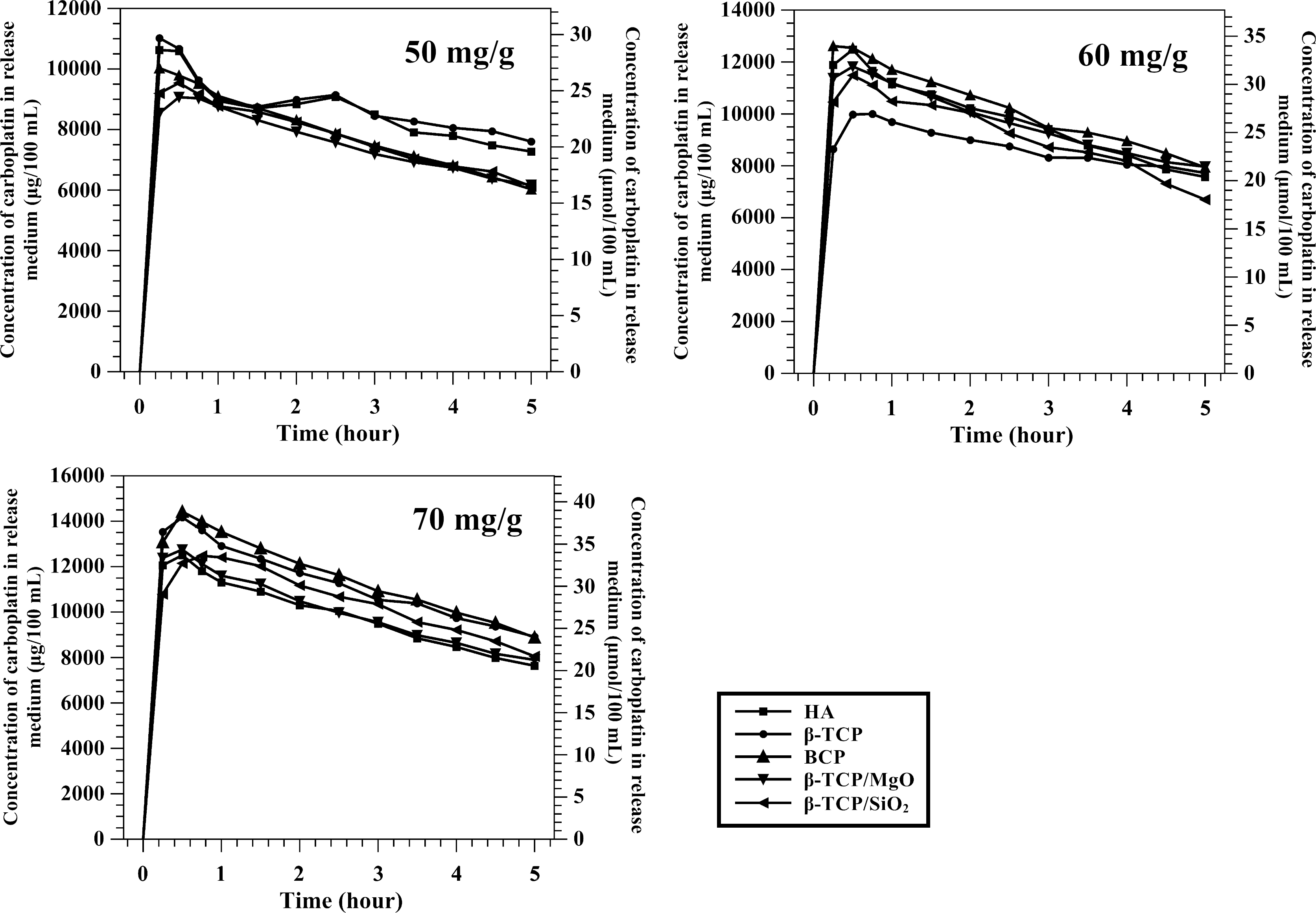

Supplement: S6 Fig — Amount of carboplatin released in μg and μmol for 250 mg of the biomaterials-carboplatin loading. (TIF) [file pone.0242565.s011.tif]
